# Statistical analysis plan for the Healthy Hands Project; single centre cluster-randomised clinical trial of a skin care program for the prevention of contact dermatitis in health care workers

**DOI:** 10.1186/s13063-018-2703-7

**Published:** 2018-08-06

**Authors:** Maryam Soltanipoor, Sanja Kezic, Judith Sluiter, Rebecca Holman

**Affiliations:** 1 Coronel Institute of Occupational Health, Amsterdam Public Health Research Institute, Academic Medical Center (AMC), Meibergdreef 9, 1105 AZ Amsterdam, The Netherlands; 2 Clinical Research Unit, Academic Medical Centre, PO Box 22660, 1100 DD Amsterdam, The Netherlands

**Keywords:** HCW, Occupational contact dermatitis, Skin care, RCT, Intervention, Statistical analysis plan

## Abstract

**Background:**

The Healthy Hands Project (HHP) is a randomised clinical trial aiming to determine the effectiveness of an intervention program in the prevention of hand dermatitis in healthcare workers (HCWs). The intervention is comprised of placing dispensers with hand creams on wards combined with continuous electronic monitoring of cream consumption and regular feedback to HCWs. The clinical severity (HECSI score) was used as the primary outcome and natural moisturising factor (NMF) levels as the secondary outcome. The study protocol for the cluster-randomised controlled trial of HHP was published in *Trials* in 2017. This article describes the detailed statistical analysis plan for the HHP trial.

**Methods/design:**

The HHP is a single-centre, cluster-randomised controlled trial with two parallel groups and blinded outcome assessment. This update article presents (1) the descriptive statistics of the primary and secondary outcomes, (2) the statistical models used for the analysis of the main outcomes, (3) sensitivity analyses on the effect of observed exposure to wet work, (4) handling of missing data including sensitivity analysis and (5) an updated power calculation. This statistical analysis plan was written prior to unblinding of the study.

**Discussion:**

This paper presents a comprehensive statistical analysis plan for the data resulting from the HHP trial. It supports transparency in reporting by clarifying differences between the previously published protocol and the proposed actual statistical analyses.

**Trial registration:**

Netherlands Trial Register (NTR), identification number NTR5564. Registered on 2 November 2015.

## Background

Healthcare workers (HCWs) have increased risk of developing occupational hand dermatitis (HD) due to frequent exposure to ‘wet work’ [1, 2]. Wet work, defined as unprotected exposure to humid environments and water; high frequency of hand-washing procedures or prolonged glove occlusion, is believed to cause irritant contact dermatitis in a variety of occupations [2, 3].

Amongst HCWs, nurses run the highest risk of HD, with an estimated point prevalence of 12 to 30% [2, 4, 5]. Almost 60% of sick leave reported during the first year after notification of the disease is related to HD [6]. Hence, HD represents a significant burden for affected individuals as well as for society. The annual costs due to occupational skin diseases for medical care, absenteeism and disability pensions are estimated at €98 million in the Netherlands [2, 7].

In the Netherlands, the Dutch Society of Occupational Medicine (NvAB) established a guideline for the prevention of occupational hand dermatitis (OHD) in 2006 [2, 8]. This guideline emphasises the importance of the skin barrier for the prevention of HD and recommends regular use of skin care products such as emollients and ointments. Consistently, in a more recent update of contact dermatitis guidelines issued by The Netherlands Society of Dermatology and Venereology (NVDV) [9], regular use of emollients to help prevent irritant contact dermatitis has been recommended [10–12].

Several skin care programmes have been introduced in the healthcare setting to help prevent occupational skin diseases [2, 13, 14]. The effectiveness of these programs seems to depend on several factors: (1) the effectiveness of protective measures (i.e. use of skin care products), (2) the adherence to these measures (i.e. frequency of application) and (3) the effectiveness of education on preventive behaviour (by raising awareness about the risk factors for HE and the importance of protective measures) [2, 15]. To avoid skin dryness and improve skin barrier function a variety of moisturisers and emollients have widely been used. The effect of skin care products on the skin barrier has mainly been investigated in experimental irritation studies [16, 17], and randomised controlled studies in HCW are scarce. With respect to the effectiveness of education, several intervention studies have shown moderate evidence that education influences behaviour, leading to a reduction in skin symptoms [18]. Regarding adherence, despite evidence from intervention studies that skin care is effective in prevention of HD, the adherence to these preventive measure in the workplace remains low [19].

Monitoring and feedback is a widely used behavioural strategy to improve adherence [2, 20]. The effects depend largely on the way that the intervention is designed and delivered [21]. Recently, an electronic monitoring system has been developed for the continuous registration of hand cream consumption. This system enables a detailed feedback to HCWs on the frequency and moments when the cream is used. In hand hygiene studies, a similar monitoring system was shown to improve compliance by 42% [2, 21].

As previously described in the published protocol [2], the Healthy Hands Project (HHP), a single-centre cluster-randomised controlled trial (RCT) investigated whether an intervention program, based on the provision of hand creams and regular feedback on cream consumption, can improve the skin condition of the hands of nurses engaged in wet work, when compared to a ‘care as usual’ control group. The intervention comprised of the provision of hand cream dispensers on the wards, electronic monitoring of their use, and repeated feedback to the HCW. Randomisation to the intervention program or care as usual was at the ward level. The effectiveness of the program will be assessed by measuring hand skin condition in workers before and after the study period. This trial has been registered in the Netherlands Trial Register with identification number NTR5564. In this paper, we will provide an update on the status of the trial and present a detailed description of the proposed data analysis. This statistical analysis plan (SAP )was written and submitted before the researcher analysing the data (MS) was un-blinded to the treatment allocation.

## Primary and secondary objectives

The primary objective of the HHP trial was to examine whether the provision of a skin care programme for the prevention of HD improves the hand skin condition of HCWs engaged in wet work. Hand skin condition was assessed using the Hand Eczema Severity Index (HECSI) score [22]. The HECSI score grades the intensity of erythema, induration, papules, vesicles, fissures, scaling and oedema for five areas of each hand (fingertips, fingers, palms, back of hands, wrists) on a scale from 0 (not present) to 3 (severe). The extent of affected skin in each area is graded from 0 to 4. The total score is obtained by multiplying the intensity with the extent and ranges from 0 to 360 points. The primary outcome focusses on the change in HECSI score between baseline and after 12 months.

The secondary outcome is the change in levels of natural moisturising factors (NMFs) in the uppermost layers of the skin, as a marker of early signs of barrier damage [23].

## Methods/design

The HHP trial was a single-centre, cluster-randomised, open, blinded-endpoint, parallel-group trial conducted on the wards of one University Medical Center in the Netherlands. This academic hospital has a total of 45 departments of which approximately 20 are clinical wards. Between 2 May and 14 June 2016, a total of 504 nurses on 19 wards were enrolled. The participants in this trial were randomised to the intervention or care as usual group at the ward level. Wards were randomised, two at a time, in a 1:1 ratio stratified according to wet work exposure (high or low) using fixed blocks of size 2. Wet work exposure was estimated at the ward level from internal purchases of soap in the period January 2016 to May 2016. Nurses and some investigators (SK) were not blinded to the allocated treatment group, but the primary outcome at 12 months was assessed by an investigator blinded to treatment outcome (MS).

**Fig. 1:**
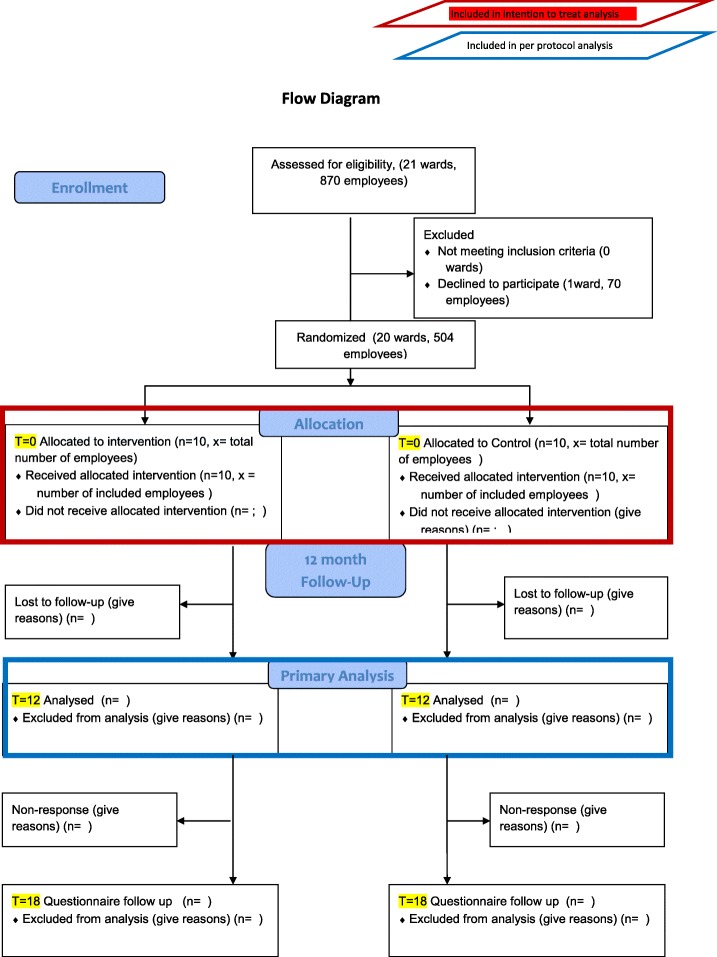
Flow diagram of the trial

This SAP focusses on the analysis of primary and secondary outcomes measured at baseline and 12 months. The extended follow-up period of 6 months (i.e. 12–18 months) stated in the flow diagram (Fig. 1) will be used to assess process outcomes, and will be out of the scope of the SAP described in the present article.

The inclusion criteria were: willing to give informed written consent; aged 18 to 65 years at the start of the study; having daily exposure to wet work activities during work; and being employed as a nurse or nutrition assistant on a participating ward. The only exclusion criteria was being employed on more than one ward.

### Intervention

The intervention comprised the provision of hand cream dispensers on the wards, electronic monitoring of their use and feedback to the HCWs. In the intervention group, the hand cream (Stokoderma® Aqua Sensitive) was provided in electronic dispensers with an electronic monitoring system on the wards at places which are easily accessible. Data on patterns of usage (frequency, timing of use, total consumption) and trends enabled structured feedback on hand cream use to the nurses and management to motivate and improve compliance. Posters, designed to present the compliance data of the cream use, were placed at the wards to remind staff of their performance and motivate to use the creams. Both the intervention and the control group received education on skin care (as stated in Table 1 of our published protocol [2] on skin protection (as care as usual) every 3 months from baseline to the end of study.

**Table 1:** Characteristics of participants in the intervention and control group

	Control Group	Intervention Group
Characteristics		
Type of ward (*n*=)		
Number of participants (*N* =)		
Sex (*n* (%) female)		
Working years (median (IQR))		
Hours worked per week (mean (SD))		
History of atopic dermatitis (*n* (%))		
Outcomes		
Median HECSI score (IQR)		
Mean NMF levels (SD)		
Frequency of use of hand alcohol (*n* (%))		
Frequency of hand washing (*n* (%))		
Frequency of glove use (*n* (%))		
Frequency use of moisturising creams (*n* (%))		

Characteristics of participants in the intervention and control group and treatment at enrolment

*HECSI* Hand Eczema Severity Index, *IQR* interquartile range, *NMF* natural moisturising factor, *SD* standard deviation

### Data collection and outcomes

The flow diagram of the study design is shown in Fig. 1. After inclusion, the participants filled in the baseline questionnaire and underwent baseline measurements (skin condition assessment by using the Hand Eczema Severity Index (HECSI) and collection of stratum corneum (SC) samples for NMF analysis). The measurements were repeated at 12 months after baseline. The primary outcome is the difference in HECSI score between baseline and 12 months. The secondary outcome is the difference in levels of NMF in the skin between baseline and 12 months.

## Statistical methods specified in the protocol

### Sample size calculation

We planned to include a total of 34 wards with on average 16 employees on each ward (a total of 544 employees) [2]. The sample size calculation was based on the expected change in the Osnabruck Hand Eczema Severity Index score between T0 and T12. A difference in the Osnabruck Hand Eczema Severity Index score of 0.4 points is regarded as clinically significant and a previous study has shown that the standard deviation of this difference is 1.2 [24]. Using a two-sided *t* test with a significance level of 0.05, degrees of freedom based on the number of wards and intra-cluster correlation of 0.05, we calculated that a study with 17 wards per treatment group and 16 employees per ward would have 81% power to detect a difference of 0.4 in group means. We based the sample size calculation on the Osnabruck Hand Eczema Severity Index rather than the HECSI score because in contrast to the former, no studies with the HECSI score in HCWs were available. This choice was supported by the fact that both scoring systems have similar content, and furthermore it has been shown that these two scores are well correlated (*r* = 0.85; *P* < 0.001) [25].

### Proposed analyses

As stated in the published trial protocol, a full SAP will be published before the researchers are unblinded [2]. We specified that we would adhere to the Consolidated Standards of Reporting Trials (CONSORT) Statement [26] and its extensions on the reporting of patient-reported outcomes in randomised trials [27] and on cluster randomised trials [28]. Due to the low-risk nature of this study, no interim analyses or safety reporting were planned and no data safety monitoring board was installed.

## Statistical analysis plan

### Sample size calculation

In the protocol, we specified that we would include 34 wards with on average 16 employees on each ward (total of 544 employees) [2]. However, we actually included 19 wards, with an average of 25 participants per ward (total 504 participants). Consequently, assuming that all participants have provided baseline and 12-month data, the power would have fallen from 81% to 66%. If, in addition, HECSI data for both baseline and 12 months would have been only available from an average of 16 participants, the power would decrease to 56%. These power calculations were performed in PASS 15 (NCSS, LLC, Kaysville, UT, USA).

### Overall principles

The data analysis will start after the 12-month follow-up data are available for all participants or it is clear that any participants, for whom no 12-month data are available, have dropped out of the study, and the study database has been cleaned and locked for this time point. All analyses will be performed by analysing participants in the trial arm, to which they were allocated in the ward level randomisation. The analyses will be first performed blinded to treatment allocation to allow the data and proposed analyses to be checked. Treatment allocation will only be unmasked when all data cleaning and analyses to be presented have been finalised.

We will present the characteristics of wards and participants using simple descriptive statistics. We will use the mean and standard deviation to describe normally distributed continuous variables and the median and upper and lower limits of the interquartile range to describe non-normally distributed continuous variables. We will assess the normality of continuous variables by visually inspecting histograms. We will use counts and percentages to present categorical variables. Two-sided *P* values of less than 0.05 will be considered statistically significant and statistical uncertainty will be expressed using two-sided 95% confidence intervals. No formal statistical testing will be performed to examine differences in baseline characteristics between the trial arms. The analyses will be performed by one of the investigators (MS) supervised by the other investigators (SK, JS) and a statistician (RH). All statistical programming and analysis will be performed using IBM SPSS statistics version 24 (IBM Corp., Armonk. NY, USA).

### Analysis populations and units

A true intention-to-treat population would include all participants randomised. However, due to substantial loss to follow-up in this study, we will perform the main analyses on a modified intention-to-treat population. This population will consist of all the participants with a HECSI score at baseline and 12 months. The per protocol population will consist of all participants with a HECSI score at baseline and 12 months and who worked in the same ward for the whole duration of the study.

### Handling of missing data

In our main analyses for the primary outcome, we will use a simple joint model approach to model the missing HECSI scores at 12 months and the observed difference between the HECSI scores at baseline and 12 months. We will perform three types of sensitivity analyses on the way that we have dealt with missing data on the primary outcome. For participants with missing HECSI score data at 12 months, we will: (1) assume the best possible outcome (HECSI score of 0); (2) assume the worst possible outcome (highest observed HECSI score) and (3) perform multiple imputation for the difference between baseline and 12-month HECSI scores. We will use on baseline characteristics as independent variables in the multiple imputations.

### List of analyses

#### Recruitment and retention and baseline characteristics

We will present the numbers of wards and employees assessed for eligibility, included, randomised to the intervention and control arms and lost to follow-up in a Consolidated Standards of Reporting Trials (CONSORT) flow diagram (see Fig. 1).

We will present the baseline characteristics of all randomised participants in each arm in a table, without performing formal statistical testing. We will present: type of ward, working years, working hours, sex, atopic tendency, self-reported HD last month, NMF level and HECSI score.

#### Deviations and violations from protocol

No major deviation or violations from protocol occurred. The main difference from protocol is the number of wards included (20 wards). while the sample size calculation was based on 34 wards.

#### Primary and secondary outcomes

We will present crude means and 95% confidence intervals for the changes in HECSI score and levels of NMF between baseline and 12 months for the intervention and control groups. In addition, we will present crude proportions of participants with both baseline and 12 months HECSI scores for both groups. We will obtain *p* values for the difference between the intervention and control groups using generalised estimating equations with an exchangeable working correlations matrix to account for clustering within wards. We will use a linear model for the changes in HECSI score and levels of NMF and a binary model with a logit link function for the missing data. We will adjust the analysis of the primary outcome for the binary factor ward-level exposure to wet work in the preceding year, used to stratify the wards in the randomisation.

#### Sensitivity analysis

In addition to the three sensitivity analyses on the handling of missing data, we will perform a sensitivity analysis on the effect of exposure to wet work observed during the study. No subgroup analyses will be performed.

## Current trial status

The HHP included 19 wards and 504 participants in the Netherlands from 2 May 2016 to approximately July 2016. The participants were followed up for 18 months. At the time of submission, the trial data are blinded to treatment allocation and the trial has not completed follow-up of the last participant (end of study planned for January 2018). The data will be cleaned and checked for completeness and internal consistency, blinded to treatment allocation. The database will only be locked after this SAP has been submitted for publication.

## Discussion

The background to, and methods for, the HHP single-centre, cluster-RCT have been previously described in the published protocol [2]. In this paper, we have provided an update on the status of the trial and presented a comprehensive SAP for the resulting data. In addition, we have clarified differences between the previously published protocol and the proposed actual statistical analyses. The principal difference from the methods presented in the protocol is the actual inclusion of 19 rather than 34 wards and 504 rather than 544 participants. Consequently, the power has fallen substantially. This paper supports transparency in reporting in the HHP by clarifying differences between the previously published protocol and the proposed actual statistical analyses.

## Abbreviations

(O)HD: (Occupational) hand dermatitis
HCW: Healthcare worker
HE: Hand eczema
HECSI: Hand Eczema Severity Index
HHP: Healthy Hands Project
METC: Medical Research Ethics Committee (MREC); in Dutch: medisch ethische toetsing commissie (METC)
NMF: Natural moisturising factor
NvAB: Netherlands Society for Occupational Health; in Dutch: Nederlandse Vereniging voor Arbeid en Bedrijfsgeneeskunde
NVDV: Netherlands Society of Dermatology and Venereology; in Dutch: Nederlandse Vereniging voor Dermatologie en Venereologie
RCT: Randomised controlled trial
SC: Stratum corneum

## References

[1] Ibler KS, Jemec GB, Flyvholm MA (2012). Hand eczema: prevalence and risk factors of hand eczema in a population of 2274 health care workers. Contact Dermatitis.

[2] Soltanipoor M, Kezic S, Sluiter JK, Rustemeyer T (2017). The effectiveness of a skin care program for the prevention of contact dermatitis in health care workers (the Healthy Hands Project): study protocol for a cluster randomized controlled trial. Trials.

[3] Fartasch M (2016). Wet work and barrier function. Curr Probl Dermatol.

[4] Visser MJ, Verberk MM, van Dijk FJ (2014). Wet work and hand eczema in apprentice nurses: part I. Contact Dermatitis.

[5] Smit HA, Burdorf A, Coenraads PJ (1993). Prevalence of hand dermatitis in different occupations. Int J Epidemiol.

[6] Diepgen TL, Agner T, Aberer W (2007). Management of chronic hand eczema. Contact Dermatitis.

[7] Van Gils RF, van der Valk PGM, Bruynzeel DJ, Coenraads PJ, Boot CRL, Anema JR (2011). Prevention of occupational hand eczema. A systematic review Contact Dermatitis.

[8] Jungbauer FH, Piebenga WP, ten Berge EE. Prevention of contact dermatitis: a national guideline. Dutch Society of Occupational Health (NvAB); 2006.

[9] Netherlands Society for Dermatology and Veneorology. Contact dermatitis: a national guideline. Netherlands Society of Dermatology and Venereology (NVDV); 2013.

[10] Williams C, Wilkinson SM, McShane P (2009). A double-blind, randomized study to assess the effectiveness of different moisturizers in preventing dermatitis induce by hand washing to stimulate healthcare use. Br JDermatol.

[11] Kütting B, Baumeister T, Weistenhöfer W (2010). Effectiveness of skin protection measures in prevention of occupational hand eczema: results of a prospective randomized controlled trial over a follow-up period of 1 year. Br J Dermatol.

[12] Kampf G, Ennen J. Regular use of a hand cream can attenuate skin dryness and roughness caused by frequent hand washing. BMC Dermatol. 2006;6:1. 10.1186/1471-5945-6-1. 10.1186/1471-5945-6-1 PMC1397860 16476166

[13] Dulon M, Pohrt U, Skudlik C, Nienhaus A (2009). Prevention of occupational skin disease in geriatric nurses. Br J Dermatol.

[14] Ibler KS, Jemec GB, Diepgen TL (2012). Skin care education and individual counselling versus treatment as usual in healthcare workers with hand eczema: randomised clinical trial. BMJ.

[15] Kütting B, Drexler H (2003). Effectiveness of skin protection creams as a preventive measure in occupational dermatitis: a critical update according to criteria of evidence based medicine. Int Arch Occup Environ Health.

[16] Held E, Lund H, Agner T (2001). Effect of different moisturizers on SLS-irritated human skin. Contact Dermatitis.

[17] Loden M (1997). Barrier recovery and influence of irritant stimuli in skin treated with a moisturizing cream. Contact Dermatitis.

[18] Held E, Wolff C, Gyntelberg F, Agner T (2001). Prevention of work-related skin problems in student auxiliary nurses: an intervention study. Contact Dermatitis.

[19] Weisshaar E, Radulescu M (2005). Skin protection and skin disease prevention courses for secondary prevention in healthcare workers: first results after two years of implementation. J Dtsch Dermatol Ges.

[20] Jamtvedt G (2006). Audit and feedback: effects on professional practice and health care outcomes. Cochrane Database Syst Rev.

[21] Ivers N (2012). Audit and feedback: effects on professional practice and healthcare outcomes. Cochrane Database Syst Rev.

[22] Held E, Skoet R, Johansen JD (2005). The Hand Eczema Severity Index (HECSI): a scoring system for clinical assessment of hand eczema. A study of inter and intraobserver reliability. Br J Dermatol.

[23] Rawlings AV, Harding CR (2004). Moisturization and skin barrier function. Dermatol Ther.

[24] Skudlik C, Dulon M, Pohrt U (2006). Osnabrueck Hand Eczema Severity Index—a study of the interobserver reliability of a scoring system assessing skin diseases of the hands. Contact Dermatitis.

[25] Duman M, Uzunali E (2015). Clinical assessment of the severity of chronic hand eczema: correlations between six assessment methods. Eur Res J.

[26] Wager L (2010). CONSORT Statement 2010. Lancet.

[27] Schulz KF, Altman DG, Moher D, for the CONSORT Group (2010). CONSORT 2010 Statement: updated guidelines for reporting parallel group randomised trials. BMJ.

[28] Campbell MK, Piaggio G, Elbourne DR, for the CONSORT Group (2012). CONSORT 2010 Statement: extension to cluster randomised trials. BMJ.

